# Clinical Molecular and Genomic Epidemiology of *Morganella morganii* in China

**DOI:** 10.3389/fmicb.2021.744291

**Published:** 2021-09-28

**Authors:** Guoxiu Xiang, Kai Lan, Yimei Cai, Kang Liao, Mei Zhao, Jia Tao, Yi Ma, Jianming Zeng, Weizheng Zhang, Zhongwen Wu, Xuegao Yu, Yuyang Liu, Yang Lu, Caixia Xu, Liang Chen, Yi-Wei Tang, Cha Chen, Wei Jia, Bin Huang

**Affiliations:** ^1^Department of Laboratory Medicine, The First Affiliated Hospital of Sun Yat-sen University, Guangzhou, China; ^2^Translational Medicine Research Center, The First Affiliated Hospital of Sun Yat-sen University, Guangzhou, China; ^3^Department of Laboratory Medicine, Guangdong Provincial Hospital of Traditional Chinese Medicine, Guangzhou, China; ^4^Department of Laboratory Medicine, General Hospital of Ningxia Medical University, Yinchuan, China; ^5^Department of Clinical Laboratory, Eye & ENT Hospital, Shanghai Medical College, Fudan University, Shanghai, China; ^6^Center for Discovery and Innovation, Hackensack Meridian Health, Nutley, NJ, United States; ^7^Department of Medical Sciences, Hackensack Meridian School of Medicine, Nutley, NJ, United States; ^8^Medical and Scientific Affairs, Cepheid, Sunnyvale, CA, United States

**Keywords:** *Morganella morganii*, molecular epidemiology, *bla*_OXA–181_, genomic island, *bla*_IMP–1_

## Abstract

**Objectives:** Ongoing acquisition of antimicrobial resistance genes has made *Morganella morganii* a new clinical treatment challenge. Understanding the molecular epidemiology of *M. morganii* will contribute to clinical treatment and prevention.

**Methods:** We undertook a 6-year clinical molecular epidemiological investigation of *M. morganii* from three tertiary hospitals in China since 2014. Antimicrobial susceptibility testing was performed using a VITEK-2 system. All isolates were screened for β-lactam and plasmid-mediated quinolone resistance genes by PCR. Isolates carrying carbapenem-resistant genes were subjected to whole-genome sequencing (WGS). The variation and evolution of these mobile genetic elements (MGEs) were then systematically analyzed.

**Results:** Among all *M. morganii* isolates (*n* = 335), forty (11.9%) were recognized as multidrug resistant strains. *qnrD1*, *aac(6′)-Ib-cr*, *bla*_TEM–104_, and *bla*_CTX–M–162_ were the top four most prevalent resistance genes. Notably, phylogenomic and population structure analysis suggested clade 1 (rhierBAPS SC3 and SC5) associated with multiple resistance genes seemed to be widely spread. WGS showed a *bla*_OXA–181_-carrying IncX3 plasmid and a *Proteus* genomic island 2 variant carrying *bla*_CTX–M–3_, *aac(6′)-Ib-cr* coexisted in the same multidrug resistant strain zy_m28. Additionally, a *bla*_IMP–1_-carrying IncP-1β type plasmid was found in the strain nx_m63.

**Conclusion:** This study indicates a clade of *M. morganii* is prone to acquire resistance genes, and multidrug resistant *M. morganii* are increasing by harboring a variety of MGEs including two newly discovered ones in the species. We should be vigilant that *M. morganii* may bring more extensive and challenging antimicrobial resistance issue.

## Introduction

*Morganella morganii* is emerging as a significant opportunistic pathogen in the hospital settings (Liu et al., 2016). A 6-year study of Gram-negative bacterial infections in Taiwan showed that this pathogen is the ninth most prevalent cause of clinical infections (Chen et al., 2012). It is reported that *M. morganii* has been involved in a variety of clinical infections, such as peritonitis, septic arthritis, sepsis, infective endocarditis (van Bentum et al., 2019) and bilateral keratitis (Zhang et al., 2017). Invasive *M. morganii* infections are usually associated with high mortality rates due to lack of appropriate empirical antibiotic treatment (Erlanger et al., 2019).

*Morganella morganii* has intrinsic resistance to ampicillin, amoxicillin and most of the first- and second-generation cephalosporins because of its intrinsic *AmpC* resistance gene (Kohlmann et al., 2018). Ongoing resistance genes or virulence factors acquisition via mobile genetic elements (MGEs) including integrative and conjugative elements (ICEs) and mobilizable genomic islands (MGIs) has promoted *M. morganii* to become a new clinical treatment challenge (Flannery et al., 2009; Schultz et al., 2017). Resistance genes were mainly plasmid mediated and harbored by various transposons or integrons, such as *bla*_KPC–2_-carrying IncP6 plasmid (Kukla et al., 2018), *bla*_OXA–181_-carrying IncN plasmid (McGann et al., 2015), *bla*_NDM–1_-carrying IncC plasmid (Aires-de-Sousa et al., 2020), *bla*_NDM–5_-carrying IncX3 plasmid (Guo et al., 2019), *bla*_IMP–27_-carrying Tn*7* transposon (Walkty et al., 2018), *bla*_CTX–M–3_-carrying Tn*6741* transposon (Luo et al., 2020), *cfr*-carrying Tn*7* transposon (Chen et al., 2019) and *bla*_GES–5_-carrying In*1390* integron (Moura et al., 2018), which have significantly contributed to the increased levels of resistance in *M. morganii*.

*Salmonella* genomic island 1 (SGI1), is an integrative MGI that has many variants (Schultz et al., 2017), and can be mobilized by IncA/C conjugative plasmids (Carraro et al., 2014). In addition, some SGI1-related elements, such as *Proteus* genomic island 2 (PGI2), *Acinetobacter* genomic island 1 (AGI1) and their variants have been described in various bacteria including diverse serovars of *S. enterica*, *Vibrio cholerae*, *P. mirabilis*, and *A. baumannii* (Cummins et al., 2020). These GIs consist of a conserved backbone and a highly genetic variable multidrug resistant region derived from one or more complex class 1 integron carrying various resistance gene cassettes (Girlich et al., 2015). The backbone usually integrates into the chromosomes at 3′ end of the *trmE* gene, and the multidrug resistant region often locates adjacent to the *res* gene (Lei et al., 2020). Noteworthy, SGI1 variant (SGI1-L) carrying resistance genes *dfrA15*, *floR*, *tetA*(G), *bla*_*CARB–2*_ and *sul1* has also been identified in *M. morganii* (Schultz et al., 2017). Besides, carbapenemase gene *bla*_NDM–1_ has also been found in the MDR region among SGI1-like sequences (Girlich et al., 2015).

Antimicrobial resistance in *M. morganii* therefore can be introduced via both resistance plasmid acquisition and genomic island horizontal transfer. However, large-scale and long-term molecular investigations of clinical isolates of *M. morganii* has rarely been conducted. The aim of this study is to conduct a clinical and molecular epidemiological investigation of *M. morganii* isolated from three tertiary hospitals in China, and to unravel the molecular mechanisms underlying antimicrobial resistance of *M. morganii* in China.

## Materials and Methods

### Bacterial Isolates

All *M. morganii* strains isolated from clinical specimen were collected from three tertiary hospitals in China from June 2014 to June 2020 including The First Affiliated Hospital of Sun Yat-sen University, Guangzhou, China (Hospital ZY), Guangdong Provincial Hospital of Traditional Chinese Medicine, Guangzhou, China (Hospital SZY) and General Hospital of Ningxia Medical University, Yinchuan, China (Hospital NX). If more than one strain were isolated from the same patient, only the first isolated strains were included. All the *M. morganii* strains were identified by VITEK-2 automatic bacterial identification system (BioMérieux, France). Isolates were cultured on Columbia agar with 5% sheep blood (BioMérieux, France) at 37°C in 5% CO_2_ atmosphere for 16 to 18 h.

### Antimicrobial Susceptibility Testing

Minimum inhibitory concentrations (MICs) of piperacillin-tazobactam, ceftriaxone, ceftazidime, cefepime, aztreonam, ciprofloxacin, levofloxacin, gentamicin, tobramycin, amikacin and trimethoprim-sulfamethoxazole were analyzed by VITEK-2 drug sensitivity analysis system (BioMérieux, France). MICs of meropenem (ApexBio, United States) and ertapenem (Menlunbio, China) were tested using micro-broth dilution method according to the guidelines of 2019 Clinical and Laboratory Standards Institute (CLSI) criteria (CLSI, 2019). Strain *Escherichia coli* ATCC 25922 was used as the quality control strain.

### Resistance Genes Detection

DNA templates were extracted by the boiling method as previously described (Sun et al., 2014). Polymerase chain reaction (PCR) was performed to detect antimicrobial resistance genes, including β-lactam resistance genes (*bla*_KPC_, *bla*_NDM_, *bla*_OXA__–48_, *bla*_VIM_, *bla*_IMP_, *bla*_CTX–M_, *bla*_TEM_, and *bla*_SHV_), and plasmid-mediated quinolone resistance (PMQR) genes [*qnrA*, *qnrB*, *qnrC*, *qnrD*, *qnrS*, *aac(6′)-Ib-cr*, *qepA*, and *oqxAB*] by using primers as described previously (Xiao-Min et al., 2014; Szabó et al., 2018; Cai et al., 2019). Primers synthesis and positive PCR products sequencing were conducted by Sangon company, Shanghai^1^. BLASTN^2^ was used to align the sequencing results. Primers and thermal conditions are presented in Supplementary Table 1.

### Conjugation Experiments

Conjugation experiment was performed to determine the transmissibility of carbapenem-resistant genes. The same amount (1 × 10^7^ CFU/mL, counted using the Sysmex UF-1000i^TM^ Automated Urine Particle Analyzer; Tokyo, Japan) of mid-logarithmic phase donor (strain zy_m28 and nx_m63) and recipient cells (*E. coli* C600) were mixed in 200 μL LB in 96-well plates. After mating for 6 h at 37°C, 20 μL mixed cultures were spread on LB agar containing 1 μg/mL meropenem plus 100 μg/mL rifampin. The conjugation frequency was calculated as transconjugants divided by number of donors. All experiments were carried out three times. Species identification, antimicrobial susceptibility testing and resistance genes detection were further performed on the transconjugants.

### Whole-Genome Sequencing, Assembly and Phylogenomic Analysis

Two strains harboring carbapenem-resistant genes were whole-genome sequenced. Genomic DNA was extracted using a MiniBEST Bacteria Genomic DNA Extraction Kit (TaKaRa, Dalian, China). Chromosomal libraries with a 300 bp insert size were prepared as previously described (Cai et al., 2019) and all barcoded libraries were sequenced on a NextSeq 500 platform (Illumina Inc., San Diego, CA, United States). To construct a current phylogenomic tree, raw reads in fastq format or pre-assembled sequences in fasta format were downloaded from NCBI database for all publicly available *M. morganii* isolates (Supplementary Figure 2). Paired-end raw reads were filtered using FASTQ preprocessor Fastp v0.12.5 to exclude library adapter and low quality reads (Chen et al., 2018) and *de novo* assembled with Unicycler v0.4.9b (Wick et al., 2017). Then, the scaffolds were annotated with Prokka v1.14.6 (Seemann, 2014). Antimicrobial resistance genes and plasmid types were identified by ABRicate v0.8.13^3^. ICEs and MGIs were identified ICEberg 2.0 (Liu et al., 2018). Pan-genome analysis was done using Roary v3.11.2 (Page et al., 2015) and core genome single-nucleotide polymorphisms (cgSNPs) were extracted using SNP-sites (Page et al., 2016). cgSNPs were filtered using VCFtools v0.1.17 (Danecek et al., 2011). After filtering, 20663 cgSNPs out of a possible 32774 cgSNPs from 1425 core orthologous genes were kept. A maximum likelihood phylogenomic tree of 246 *M. morganii* genomes was constructed by RAxML v8.2.12 (Stamatakis, 2014) using the filtered cgSNPs. Population structure based on Bayesian analysis was then identified using rhierBAPS v1.0.1 (Tonkin-Hill et al., 2018) using the filtered cgSNPs. Besides, population structure analyzed by PopPUNK is also provided as a supplement (Lees et al., 2019). The phylogenetic tree is displayed and annotated using iTOL v5 (Letunic and Bork, 2021).

### Genomic Island Assembly, Comparation and Phylogenetic Analysis

The GI was predicted by IslandViewer 4^4^. The scaffolds assembled by Unicycler v0.4.9b (Wick et al., 2017) were reordered using Ragout v2.3 (Kolmogorov et al., 2014) with reference to the most similar GI sequence recognized by BLASTN. The gaps were filled using PCR method (Primers and thermal conditions are presented in Supplementary Table 1). Then, the complete GI sequence was annotated with Prokka v1.14.6 (Seemann, 2014). To compare the variations of current SGI1-related GIs, single-copy orthogroups of GI sequence found in this study and other pre-assembled GIs from NCBI database were identified by OrthoFinder v2.3.7 (Emms and Kelly, 2019), and a maximum likelihood phylogenetic tree is then done from the concatenated alignment using RAxML v8.2.12 (Stamatakis, 2014). The phylogenetic tree is displayed and annotated using iTOL v5 (Letunic and Bork, 2021). Genetic elements of PGI2 family were visualized and compared using Easyfig v2.2.2 (Sullivan et al., 2011).

### Plasmids Assembly, Comparation and Phylogenetic Analysis

Plasmids were assembled using plasmidSPAdes (Antipov et al., 2016). The scaffolds were reordered using Ragout v2.3 (Kolmogorov et al., 2014) with reference to the most similar plasmid sequence recognized by BLASTN. The gaps were filled using PCR method (Primers and thermal conditions are presented in Supplementary Table 1). Plasmids or genetic elements visualization and comparison were conducted using gggenes^5^ and Easyfig v2.2.2 (Sullivan et al., 2011). Single-copy orthogroups of assembled plasmid and other closely related plasmids were identified by OrthoFinder v2.3.7 (Emms and Kelly, 2019), and a maximum likelihood phylogenetic tree is then done from the concatenated alignment using RAxML v8.2.12 (Stamatakis, 2014). The phylogenetic tree is displayed and annotated using Evolview v3 (Subramanian et al., 2019).

### Data Availability

Newly sequenced genomic island and plasmids were deposited in GenBank under the accession numbers, MW080367.1, MW080368.1, and MW150990.1, respectively.

### Statistical Analysis

All the statistical analysis were performed by SPSS 19.0 (IBM Corp., Armonk, United States). *Chi*-square test or Fisher’s exact test was applied to evaluate the differences of antibiotic resistance rates and resistance genes prevalence among the three hospitals. Mann–Whitney *U* rank sum test was applied to evaluate the differences of antimicrobial resistance genes distribution between different phylogenomic clades. *P* < 0.05 was considered statistically significant.

### Ethical Considerations

This study was approved by Institutional Review Board of The First Affiliated Hospital of Sun Yat-sen University. The study was retrospective and all clinical data were anonymized.

## Results

### Isolation and Characterization of *Morganella morganii*

A total of 335 *M. morganii* clinical isolates were collected from two hospitals in Guangzhou, Southeast China and one hospital in Yinchuan, Northwest China during June 2014 to June 2020. In general, urine (26.6%, 89/335), wound secretion (14.0%, 47/335), sputum (11.9%, 40/335) and shunt fluid (10.2%, 34/335) were the top four most frequent sample types of *M. morganii* clinical isolates, while the sample-type distributions among the three hospitals were statistically different (*χ*^2^ = 85.0, *P* < 0.001) (Supplementary Table 2). These strains were mainly isolated from hepatic-biliary-pancreatic surgery (14.0%, 47/335), intensive care unit (11.0%, 37/335), general surgery (9.6%, 32/335), and burn center (6.0%, 20/335) (Supplementary Table 3).

### Antibiotic Resistance Rates of *Morganella morganii*

Common antibiotics had different MIC distributions. Ceftazidime, gentamicin, and trimethoprim-sulfamethoxazole showed classic bimodal distributions. Meropenem, ertapenem, amikacin, etc. showed clear distributions for the sensitive cases. Ciprofloxacin, levofloxacin and tobramycin showed more spread distributions around all the concentration ranges (Supplementary Figure 1). The susceptibility testing results showed that 130 (38.81%), 66 (19.70%), 61 (18.21%), 55 (16.42%), and 47 (14.0%) strains were resistant to trimethoprim-sulfamethoxazole, ceftazidime, gentamicin, ciprofloxacin and ceftriaxone, respectively (Supplementary Table 4). Among them, strain zy_m28 from Hospital ZY and strain nx_m63 from Hospital NX showed intermediate resistant to meropenem (MIC = 2 μg/mL). There was no statistical difference of the resistance rates between the two hospitals in Guangzhou (Hospital ZY and Hospital SZY). However, the resistance rates of the ceftriaxone, ceftazidime, cefepime, ciprofloxacin, gentamicin, and trimethoprim-sulfamethoxazole were significantly higher in Hospital NX compared to Hospital ZY and SZY. Forty (11.9%) *M. morganii* isolates were recognized as multidrug resistance (MDR) strains as they were resistant to three or more classes of antibiotics, and the MDR *M. morganii* rates were also statistically different among the three hospitals (*χ*^2^ = 10.5, *P* < 0.05). The MDR rate in Hospital NX was higher than in Hospital ZY. The strain zy_m28 was a MDR strain resistant to ceftriaxone, ciprofloxacin, gentamicin and trimethoprim-sulfamethoxazole.

### Resistance Genes Prevalence in *Morganella morganii*

All isolates were detected by PCR for β-lactam resistance genes and PMQR genes. PCR results showed that 41 (12.2%), 27 (8.1%), 80 (23.9%), and 40 (12.0%) *M. morganii* strains carried *bla*_TEM_, *bla*_CTX–M_, *aac(6′)Ib-cr* and *qnrD1*, respectively (Table 1). Among *bla*_TEM_ and *bla*_CTX–M_, *bla*_TEM–104_ and *bla*_CTX–M–162_ were the most popular subtypes. Two carbapenem-resistant genes *bla*_OXA–181_ and *bla*_IMP–1_ were detected in strain zy_m28 and nx_m63, respectively. MDR strain zy_m28 carried *bla*_OXA–181_, *bla*_TEM–1_, *bla*_CTX–M–__3_, *qnrS1* and *aac(6′)Ib-cr* at the same time. Other PMQR genes *qnrA1*, *qnrB1*, *oqxA*, and *oqxB* were also detected. Sixty (17.9%) *M. morganii* strains carried at least one of the extended-spectrum β-lactamase (ESBL) resistance genes, and 103 (30.8%) carried at least one of the PMQR genes. There was no statistical difference in ESBL resistance genes prevalence among the three hospitals, while prevalence rates of PMQR genes among the three hospitals were statistically different (*χ*^2^ = 9.3, *P* < 0.05). PMQR genes were more prevalent in Hospital NX than in Hospital ZY and SZY. Thirty-four (10.1%) *M. morganii* strains carried both ESBL and PMQR resistance genes, and *bla*_TEM__–104_ coexistence with *qnrD1* or *aac(6′)Ib-cr* were the most common combinations (Supplementary Table 5).

**TABLE 1:** Prevalence of resistance genes in *Morganella morganii* clinical isolates.

Resistance genes	Hospital ZY (*n* = 82)	Hospital SZY (*n* = 112)	Hospital NX (*n* = 141)	Total (*n* = 335)	Three hospitals comparation	
	Prevalence (%)	Prevalence (%)	Prevalence (%)	Prevalence (%)		
*bla*_IMP_	0.0	0.0	0.7	0.3	\	
*bla*_OXA–48_	1.2	0.0	0.0	0.3	\	
*bla*_TEM_	9.8	10.7	14.9	12.2	*χ*^2^ = 1.6	*P >* 0.05
*bla*_CTX–M_	11	3.6	9.9	8.1	*χ*^2^ = 4.7	*P >* 0.05
ESBL	18.3	11.6	22.7	17.9	*χ*^2^ = 5.2	*P >* 0.05
*qnrA*	0.0	0.0	2.1	0.9	\	
*qnrB*	1.2	0.0	0.0	0.3	\	
*qnrD*	19.5	19.6	29.8	23.9	*χ*^2^ = 4.7	*P >* 0.05
*qnrS*	1.2	0.0	3.6	1.8	\	
*aac(6′)Ib-cr*	8.5	9.8	15.6	11.9	*χ*^2^ = 3.2	*P >* 0.05
*oqxA*	0.0	0.0	1.4	0.6	\	
*oqxB*	0.0	0.0	0.7	0.3	\	
PMQR^*b*,*c*^	23.2	25.0	39.7	30.8	*χ*^2^ = 9.3	*P* < 0.05
*bla*_TEM_, *qnrD*	1.2	6.3	5.7	4.8	*χ*^2^ = 3.2^a^	*P >* 0.05
*bla*_TEM_, *aac(6′)Ib-cr*	2.4	4.5	6.4	4.8	*χ*^2^ = 1.7^a^	*P >* 0.05
*bla*_CTX–M_, *qnrD*	2.4	2.7	6.4	4.2	*χ*^2^ = 2.5^a^	*P >* 0.05
*bla*_CTX–M_, *aac(6′)Ib-cr*	1.2	2.7	2.8	2.7	*χ*^2^ = 0.8^a^	*P >* 0.05
ESBL, PMQR^*b*^	6.1	7.1	14.9	10.2	*χ*^2^ = 6.1	*P* < 0.05

*ESBL, extended-spectrum *β*-lactamase; PMQR, plasmid mediated quinolone resistance. Hospital ZY, The First Affiliated Hospital of Sun Yat-sen University, Guangzhou, China; Hospital SZY, Guangdong Provincial Hospital of Traditional Chinese Medicine, Guangzhou, China; Hospital NX, General Hospital of Ningxia Medical University, Yinchuan, China.*

*^*a*^Using Fisher’s exact test.*

*^*b*^Prevalence of resistance gene in Hospital NX was higher than Hospital ZY (*chi*-square test pairwise comparison, adjusted α = 0.017).*

*^*c*^Prevalence of resistance gene in Hospital NX was higher than Hospital SZY (*chi*-square test pairwise comparison, adjusted α = 0.017).*

*\. Dissatisfaction the preconditions of *chi*-square test.*

### Conjugation Experiments

Carbapenem-resistant genes *bla*_OXA–181_ and *bla*_IMP–1_ could be successfully transferred from strain zy_m28 and nx_m63 to the *E. coli* C600. Conjugation frequencies of *bla*_OXA–181_ and *bla*_IMP–1_ plasmids were (2.4 ± 7.9) × 10^–4^ per donor cell and (1.2 ± 3.8) × 10^–4^ per donor cell, respectively. Susceptibility testing results showed that transconjugants zy_m28-*E. coli* C600 were resistant to meropenem, ciprofloxacin, and transconjugants nx_m63-*E. coli* C600 were resistant to meropenem (Supplementary Table 6). Transconjugants zy_m28-*E. coli* C600 were found to contain *bla*_OXA–181_, *qnrS1* but without *bla*_TEM–1_, *bla*_CTX–M–__3_ and *aac(6′)Ib-cr*. Transconjugants nx_m63-*E. coli* C600 were found to contain *bla*_IMP–1_ as expected. Conjugative transfer of carbapenem-resistant genes *bla*_OXA–181_ and *bla*_IMP–1_ could be a threat to public health worldwide.

### Phylogenomic Analysis and Population Structure of *Morganella morganii*

**FIGURE 1:**
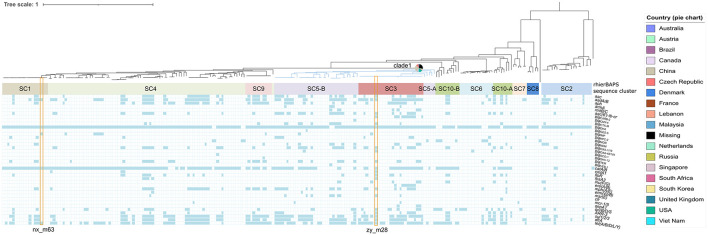
Phylogenomic and population structure analysis of *Morganella morganii* genomes worldwide combined with the distribution of resistance genes among *Morganella morganii.* Isolates identifiers are omitted. Branch in blue shows phylogenomic clade 1. Pie chart beside the blue branch represents the geographical origins of clade 1 isolates. BAPS sequence clusters are indicated by filled colored rectangles. Strain zy-m28 and nx_m63 in this study are noted with orange hollow rectangles.

Two strains zy_m28 (GCA_014333515.1) and nx_m63 (GCA_014283905.1) harboring carbapenem-resistant genes were whole-genome sequenced. Other *M. morganii* genomes were accessed from NCBI assembly database and SRA database (Supplementary Figure 2). A total of 20663 filtered cgSNPs were generated by Roary v3.11.2 (Page et al., 2015). Phylogenomic analysis of the two and current public *M. morganii* genomes showed that these *M. morganii* clustered into multiple clades. It is interesting that clade 1 (showed in blue) associated with multiple resistance genes containing *bla*_KPC–2_, *bla*_IMP–10_, *bla*_IMP–27_, *bla*_OXA–48_, *bla*_OXA–181_, *bla*_NDM–1_, *bla*_NDM–7_, *bla*_GES–5_, *mcr-1*, and *mcr-5*, seemed to be widely spread in multiple global regions, including Asia, North America, South America, Europe, Australia and South Africa (Figure 1 and Supplementary Table 7). Mann–Whitney *U* rank sum test showed that antimicrobial resistance genes (ARGs) in clade 1 was more than ARGs in non-clade 1 (*P* < 0.001) (Supplementary Figure 3A). Some noticeable MGEs, such as *bla*_KPC–2_-carrying IncP-6 plasmid, *bla*_NDM–1_-carrying IncN2 plasmid, *bla*_NDM–7_-carrying IncX3 plasmid, *bla*_OXA–48_-carrying IncL/M plasmid, *bla*_KPC–2_-carrying IncR plasmid, *mcr-1*-carrying IncX4 plasmid, and MGI*VflInd1* were identified in *M. morganii* for the first time in this interesting clade (Supplementary Tables 7, 8). rhierBAPS population structure analysis showed that *M. morganii* could be classified into ten sequence clusters (SCs) based on the cgSNPs. The interesting clade 1 associated with multiple resistance genes (Figure 1, blue clade) was classified as SC3 and SC5. PopPUNK population structure analysis showed that clade 1 could be classified as combined cluster 2, 7, 8, and 12 (Supplementary Table 7). PopPUNK offered more detailed population structure groups than rhierBAPS, which may be resulted from PopPUNK considered both the accessory distance and core distance (Supplementary Figure 3B). MDR strain zy_m28 was classified as SC3. Growth curve showed its greater fitness advantage in LB medium and M9CA minimal medium than strain nx_m63 classified as SC1 and larger maximum population capacity (*Ym*) than a plasmid-free control strain zy_m3 (Supplementary Figures 3C,D). There was no statistical relationship between population structure and geographical origin (*χ*^2^-test).

### Genetic Contexts of Mobile Genetic Elements in *Morganella morganii* Strain zy_m28

Strain zy_m28 was isolated from the right pelvic drainage fluid of a 28-year-old Korea patient with Crohn’s disease and intestinal infection, and *Enterococcus raffinosus* strain was also cultured from the same sample. WGS analysis showed that two MGEs, PGI2 variant PGI2-zym28 (MW080367.1) and *bla*_OXA–181_-carrying IncX3 plasmid pZYM28-OXA-181 (MW080368.1) coexisted in zy_m28.

**FIGURE 2:**
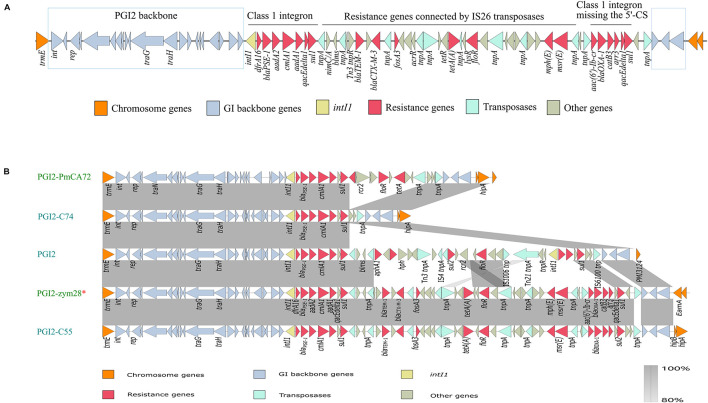
**(A)** Genetic structure of PGI2-zym28. The different regions corresponding to class 1 integrons and other regions are indicated on the horizontal line. **(B)** Genetic context comparisons of GIs in PGI2 family. Accession numbers of these GIs are MH990678.1, MK847916.1, MG201402.1, MW080367.1, and MK847915.1 from top to bottom. All the GIs are from *Proteus mirabilis* except PGI2-zym28. GIs identified from animal hosts are shown in blue and from *Homo sapiens* are shown in green. PGI2-zym28 in this study is labeled with a red *. Genes and ORFs are shown as arrows, which indicate their orientations of transcription. Shared regions with 80–100% identity are indicated by gradual shading. The picture was drawn with Easyfig v2.2.2.

Bioinformatics analysis showed that PGI2-zym28 integrated into the chromosome between the *trmE* and EamA-like transporter family genes. The MDR region in PGI2-zym28 contained one complete class 1 integron carrying *dfrA16*, *bla*_*PSE–1*_, *aadA2*, *cmlA1* and *aadA1* gene cassettes at the left side, and one partial class 1 integron missing the 5′-CS carrying *aac(6′)-Ib-cr*, *bla*_OXA–1_, *catB3* and *arr-3* gene cassettes at the right side, as well as various additional resistance genes including *bla*_TEM–1_, *bla*_CTX–M–3_, *fosA3*, *tet(A)*, *floR*, *mph(E)* and *msr(E)* connected by IS*26* (Figure 2A).

To compare the variations of current SGI1-related GIs, we then compared the genetic structure and conducted a phylogenetic analysis for SGI1-like sequences (Supplementary Figure 4). In general, these GIs could be divided into three clades related to SGI1 family, AGI1 family and PGI2 family, respectively. The backbone regions in all GIs were relatively conservative, however, deletion, insertion and inversion events still happened (SGI1 family). Notably, most SGI1-like sequences were found in *S. enterica* and *P. mirabilis*, but it has now also been described in *A. baumannii*, *E. coli* and *E. cloacae* (AGI1 and PGI2 family). PGI2 family showed significant variations in MDR regions, bringing challenges to the control of antimicrobial resistance (Figure 2B). PGI2-zym28 showed high identities with PGI2-C55 of animal origin (MK847915.1), suggesting horizontal transfer of PGI2 variants facilitated the dissemination of antimicrobial resistance.

**FIGURE 3:**
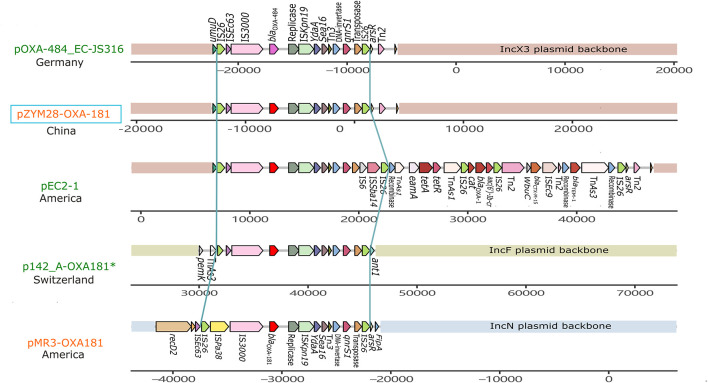
Genetic context comparisons among the transposons carrying *bla*_OXA–181_ and *qnrS* in different plasmids. Genes and ORFs are shown as arrows without orientations. Accession numbers of these plasmids are CP058621.1, MW080368.1, CP041956.1, CP048338.1, and KM660724.1 from top to bottom. All the plasmid are isolated from *Homo sapiens* except p142_A-OXA-181 (labeled with a green *). Plasmids identified from *Morganella morganii* are shown in orange and from *Escherichia coli* are shown in red. pZYM28-OXA-181 in this study is noted with a blue hollow rectangle. The picture was drawn with gggenes aligned with *bla*_OXA–181_.

pZYM28-OXA-181 was an IncX3 type plasmid carrying *bla*_OXA–181_ and *qnrS1*. Resistance genes *bla*_OXA–181_ and *qnrS1* were flanked by two same oriented IS*26*. The same structure was also found in IncF and IncN plasmids (Figure 3). Though the fragment (IS*26*-IS*3000-bla*_OXA–181_-IS*Kpn19*-Tn*3-qnrS*-IS*26*) was relatively conservative, variations still happened. In plasmid pOXA-484_EC-JS316 (CP058621.1), *bla*_OXA–181_ was replaced with *bla*_OXA–484_. While in plasmid pEC2-1 (CP041956.1), the transposon was flanked by IS*6* on the 3′ side, joining *aac(6′)-Ib-cr*, *bla*_CTX–M–3_ and *bla*_TEM–1_ with the help of some transposons.

**FIGURE 4:**
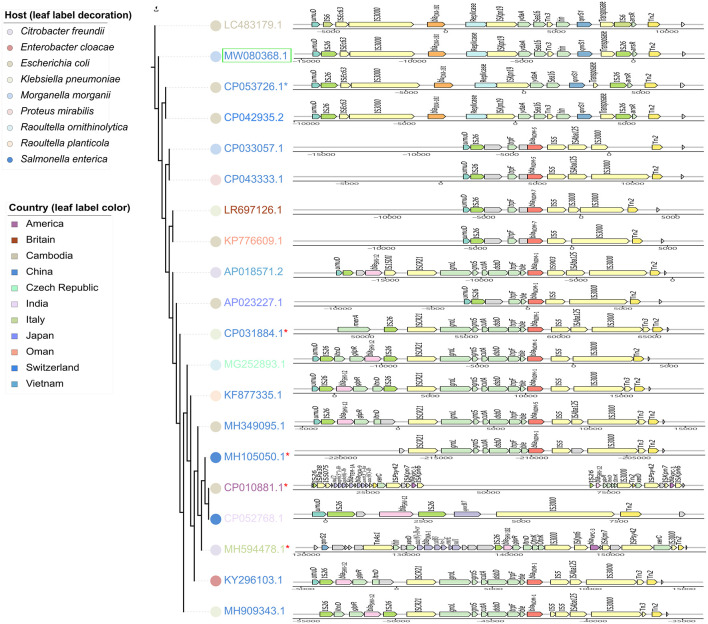
Phylogenetic analysis of IncX3 plasmids combined with the comparisons of their resistance regions. Branch length represents genetic variabilities. Genes and ORFs are shown as arrows without orientations. The colors of symbols beside leaf labels indicate bacterial hosts of these plasmids and leaf label colors represent geographic origins. pZYM28-OXA-181 in this study is noted with a green hollow rectangle. Cointegrate plasmids consisting of IncX3 and other type of plasmid backbones are noted with red *. The resistance regions comparisons were drawn with gggenes aligned with *bla*_NDM_.

IncX3 plasmids are prevalent worldwide (Roer et al., 2018). Genetic structures of IncX3 plasmids in different species were compared, and a phylogenetic tree was also constructed. Phylogenetic tree of IncX3 plasmids showed minor genetic variabilities. There were three main types of transposons disseminated in IncX3 type plasmids, with one mainly associated with *bla*_NDM_, one with *bla*_OXA–181_, and the other linked to *bla*_KPC_ and ESBL-encoding genes (Figure 4). *bla*_NDM_ genes were located on Tn*125*-like transposons (IS*CR21-groL-groS-cutA-dsbD-trpF*), while *bla*_OXA–181_ genes were located on the IS*26*-flanked transposons as described above. These transposons always integrated into the IncX3 backbone adjacent to *umuD* gene and flanked by Tn*2* at the other side. IncX3 plasmids harboring carbapenemase genes were frequently described in different species, suggesting it is an important medium for the spread of carbapenemase genes. This poses a challenge for antimicrobial resistance control.

### Genetic Context of Mobile Genetic Elements in *Morganella morganii* Strain nx_m63

Strain nx_m63 was recovered from the urine of a 57-year-old Chinese patient with deep vein thrombosis and urinary tract infection, and a MDR *E. coli* strain was also cultured from the same sample. WGS analysis showed that nx_m63 harbored a *bla*_IMP–1_-carrying IncP-1β type plasmid pNXM63-IMP (MW150990.1).

**FIGURE 5:**
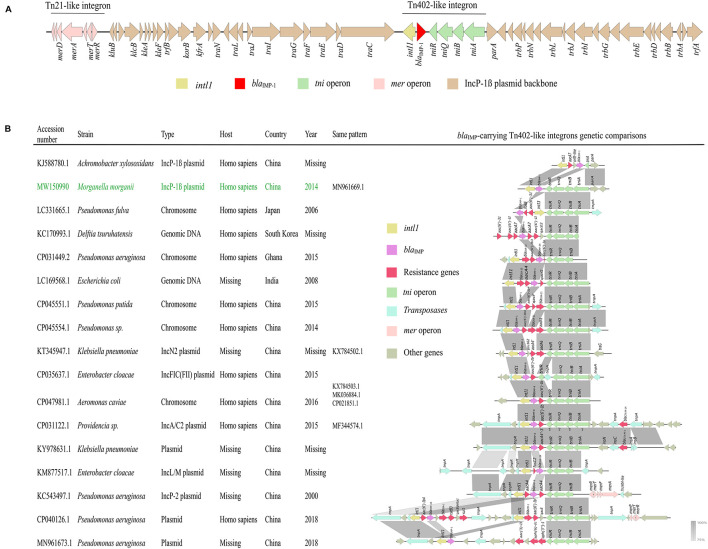
**(A)** Genetic structure of pNXM63-IMP. The region of Tn*402*-like integron is indicated on the horizontal line. **(B)** Genetic context comparisons among *bla*_IMP_-carrying Tn*402*-like type 1 integrons. Other information, such as accession number, strain, sequence type, host, country, year and sequences with the same genetic pattern are also shown. The information of the Tn*402*-like type 1 integron found in pNXM63-IMP in this study is shown in green. Genes and ORFs are shown as arrows, which indicate their orientations of transcription. Shared regions with 75–100% identity are indicated by gradual shading. The picture was drawn with Easyfig v2.2.2.

pNXM63-IMP carried both Tn*402*-like integron and Tn*21*-like integron that were inserted into the downstream of *traC2* and *trfA*, respectively. *bla*_IMP–1_ was located on the Tn*402*-like type 1 integron without accompanying other gene cassettes (Figure 5A). We then compared the genetic structure of current *bla*_IMP_-carrying Tn*402*-like integrons and found this type of integrons could both integrate into chromosomes and plasmids in various species (Figure 5B). In comparison to a similar *bla*_IMP–1_-carrying IncP-1β plasmid pA22732 (KJ588780.1), the *tni* operon of the Tn*402*-like type 1 integron in pNXM63-IMP was complete. Except for *bla*_IMP–1_, other resistance genes, including ESBL gene *bla*_CTX–M–14_ (CP031122.1) and carbapenemase gene *bla*_VIM–1_ (CP040126.1) have also been found in Tn*402*-like type 1 integrons. We should closely monitor these flexible carriers of resistance genes.

## Discussion

In this study, we collected all the *M. morganii* strains isolated from June 2014 to June 2020 in three tertiary hospitals in China, regardless of sample types and department sources, to provide an overview of molecular epidemiology of *M. morganii*. The antibiotic resistance rates and prevalence of resistance genes from Hospital NX in Northwest China were generally higher than those of Hospital ZY and SZY in Southeast China, which may be resulted from the differences in economic and medical conditions, prescribing behaviors and differences in knowledge of antimicrobial clinicians (Zhen et al., 2019). We also found that PMQR and ESBL-encoding genes usually coexisted, causing multidrug resistance, consistent with the research of Briales et al. (2012). Conjugation experiment confirmed the co-transfer of *bla*_OXA–181_ and *qnrS1* in our study.

Phylogenomic and population structure analysis of public *M. morganii* genomes showed that clade 1 (rhierBAPS SC3 and SC5) associated with multiple resistance genes seemed to be widely spread in multiple global regions. The resistance genes pattern of this clade was largely divergent from other clades. Various putative IMEs or ICEs, and noticeable carbapenem and polymyxin -resistant plasmids were identified in the clade. Growth curve showed that strain zy_m28 belonging to rhierBAPS SC3, had greater fitness advantage than strain nx_m63 belonging to rhierBAPS SC1 and even had larger maximum population capacity (*Ym*) than a plasmid-free control strain zy_m3. A reasonable guess is that these subclusters may be more suitable hosts to hold acquired resistance genes than other clusters because of low fitness cost and may cause resistance issue in the future. Further studies and more samples are needed to demonstrate this hypothesis.

Mobile genetic elements are essential in horizontal transfer of resistance genes. In this study, we identified a PGI2 variant PGI2-zym28 carrying *bla*_CTX–M–3_ and *aac(6′)-Ib-cr*. To the best of our knowledge, this is the first report of PGI2 variant in *M. morganii*. PGI2-zym28 showed high identities with PGI2-C55 initially found in a *P. mirabilis* strain isolated from a Chicken in 2018 from Shandong, China (Lei et al., 2020). The result suggests that PGI2-like sequences may be transferred horizontally between *P. mirabilis* and *M. morganii* among humans and animal sources. Further studies are needed to monitor the spread of PGI2-like sequences in clinical settings and understand their effects on clinical antimicrobial resistance.

An IncX3 plasmid pZYM28-OXA-181 harboring *bla*_OXA–181_ and *qnrS1* was found to coexist with PGI2-zym28 in strain zy_m28. *bla*_OXA–181_, a variant of *bla*_OXA–48_ family, is showing an increasing prevalence since first reported in India in 2007 (Qin et al., 2018). While *bla*_OXA–181_ has been found in different plasmid types, such as IncN (McGann et al., 2015), IncT (Villa et al., 2013) and IncX3 (Qin et al., 2018), *bla*_OXA–181_ -harboring IncX3 plasmids are the most prevalent (Roer et al., 2018; Feng et al., 2019). There are two main epidemic types of IncX3 plasmids, one spreading *bla*_OXA–181_ and *qnrS* together on an IS*26*-flanked composite transposon and the other spreading *bla*_NDM_ and *bla*_SHV–12_ together on a Tn*125*-like transposon. To the best of our knowledge, this is the first report of IncX3 type plasmid carrying *bla*_OXA–181_ in *M. morganii*. The spread of *bla*_OXA–181_-harboring IncX3 plasmid in clinical *M. morganii* strains may further limit clinical therapeutic solutions.

Another noticeable plasmid, an IncP-1β type plasmid pNXM63-IMP carrying *bla*_IMP–1_ on a Tn*402*-like class 1 integron, was found in the *M. morganii* strain nx_m63 for the first time. The Tn*402*-like integrons carrying *bla*_IMP_ were found in multiple species including *P. aeruginosa* (Xiong et al., 2013), *E. cloacae* (Wang et al., 2015), and *K. pneumoniae* (Jiang et al., 2017). Interestingly, they were often reported in Asia especially in China. Although the *bla*_IMP–1_-carrying Tn*402*-like integron found in this study did not carry other resistance gene cassettes, it is worth noting that with the help of various transposases, more complex multidrug resistant Tn*402*-like integrons carrying both *bla*_IMP–45_ and *bla*_VIM–1_ are emerging (unpublished data, CP040126.1).

This study demonstrates that a clade of *M. morganii* is prone to acquire resistance genes, and multidrug resistant *M. morganii* are increasing by harboring a variety of MGEs including two newly discovered ones in the species. The variation and evolution of these MGEs may bring more extensive and challenging antimicrobial resistance issue. Horizontal infection control strategies should be considered in tackling antimicrobial resistance in *M. morganii* and other pathogens.

## Data Availability Statement

The original contributions presented in the study are publicly available. This data can be found here: https://www.ncbi.nlm.nih.gov/genbank/, under the accession numbers, MW080367.1, MW080368.1, and MW150990.1.

## Author Contributions

BH, WJ, CC, and KLi designed the experiments. GX, YC, MZ, JT, YM, WZ, ZW, XY, and YLi collected the isolates. GX, YC, and YM completed the experiments. GX, YC, and JZ completed the data analysis. GX, KLa, YC, and KLi completed the draft of the manuscript. YL, CX, LC, and Y-WT revised the manuscript. All authors contributed to the article and approved the submitted version.

## Conflict of Interest

The authors declare that the research was conducted in the absence of any commercial or financial relationships that could be construed as a potential conflict of interest.

## Publisher’s Note

All claims expressed in this article are solely those of the authors and do not necessarily represent those of their affiliated organizations, or those of the publisher, the editors and the reviewers. Any product that may be evaluated in this article, or claim that may be made by its manufacturer, is not guaranteed or endorsed by the publisher.

## Footnotes

1
https://www.sangon.com/

2
www.ncbi.nlm.nih.gov/blast/

3
https://github.com/tseemann/abricate

4
http://www.pathogenomics.sfu.ca/islandviewer/

5
https://github.com/wilkox/gggenes
