# Supplementary material for: Clinical Molecular and Genomic Epidemiology of Morganella morganii in China
Source: Front Microbiol. 2021 Sep 28;12:744291. doi: 10.3389/fmicb.2021.744291 (PMC8507844; doi:10.3389/fmicb.2021.744291)
Supplement: Supplementary file 1 [file Data_Sheet_1.docx]

**Supplementary Materials and Methods**

**Growth curve**

The growth curve was studied by inoculating 100 μL overnight culture of *Morganella morganii* strains belonging to different BAPS subclusters in 6 mL fresh LB broth or M9CA minimal medium, and incubating at 37°C, 260rpm on a shaker. Bacterial growth was recorded by monitoring OD600 every 1 h for 14 h to 24 h at 37°C. Experiments were repeated in three separate assays. The best growth models were determined according to the bigness of R-squared values. Squared Euclidean distances were calculated to measure the similarity between growth curves. For curves with the same time length, calculate the distance between every two points and then sum them. Similarity is inversely proportional to distance. The random error distance is set at within 20% of the control growth curve. If the distance between two curves is greater than the random error distance, it is considered that there is a difference between the two curves.

**Supplementary Figure Legends**

**Figure S1.** MIC distributions of *Morganella morganii* clinical isolates.

**Figure S2.** The composition of *Morganella morganii* genomes used for phylogenomic analysis.

**Figure S3.** **(A)** Box plot of antimicrobial resistance genes distribution in different phylogenomic clades. Differences in characteristics between groups were analyzed using Mann-Whitney U rank sum test. **(B)** Population structure of *M. morganii* isolates by PopPUNK analysis. The picture was drawn by GrapeTree. Each circle represents an isolate and each line is a branch. Branch length varies on the distance between isolates. **(C, D)** Growth curve of *Morganella morganii* strains in LB medium or M9CA minimal medium. The colored smooth curves represent the fitted growth curves. The meaning of each parameter is as follows. *Y0*: starting population; *Ym*: maximum population; *K*: rate constant; *R squared*: *R^2^* of fitted curve. *dis(a,b)*, the Euclidean distance between a and b. Strain zy_m3 is plasmid-free and serves as a control. Strain zy_m28 harboring *bla*_OXA-181_-carrying IncX3 plasmid belongs to BAPS sequence cluster 3 (SC3). Strain nx_m63 harboring *bla*_IMP-1_-carrying IncP-1β plasmid belongs to BAPS sequence cluster 1 (SC1).

**Figure S4.** Phylogenetic analysis of *Salmonella* related genomic islands combined with genetic context comparisons. Branch symbols represent the geographic origins. The color of dashed branches represents the isolation source. Bacterial hosts are indicated by the color of leaf labels. PGI2-zym28 in this study is noted with a yellow hollow rectangle. Filled dark gray rectangles represent genomic island backbones sharing more than 80% similarity to SGI1 backbone, while filled light gray rectangles represent genomic island backbones sharing less than 80% similarity to SGI1 backbone. Filled blue rectangles represent transposase insertion. Filled yellow rectangles represent inversion events. White rectangles represent deletions. Antimicrobial resistance genes are shown with filled red triangles.


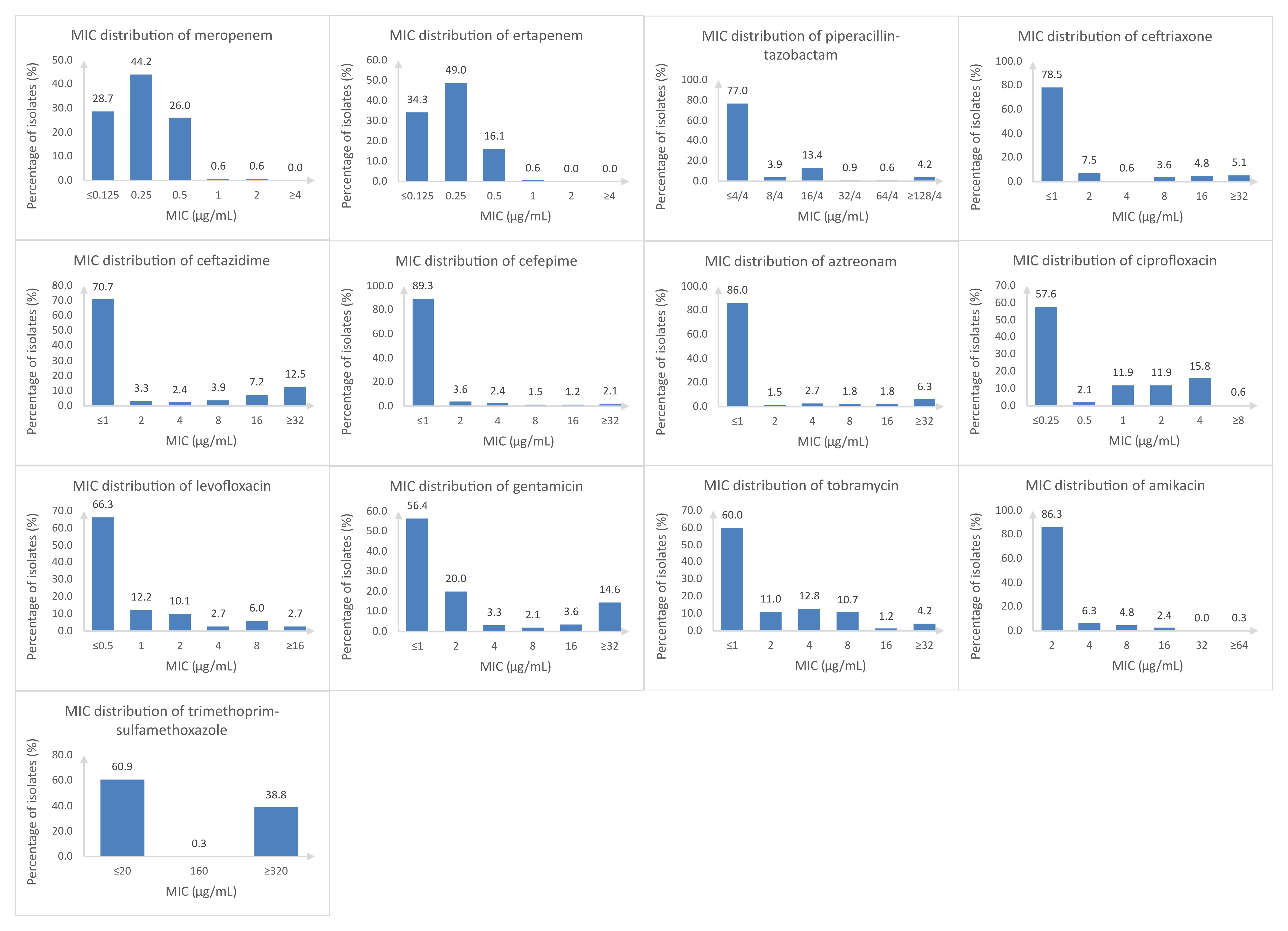


**Figure S1** MIC distributions of *Morganella morganii* clinical isolates.

**

**

**Figure S2** Composition of *Morganella morganii* genomes used for phylogenomic analysis.

**
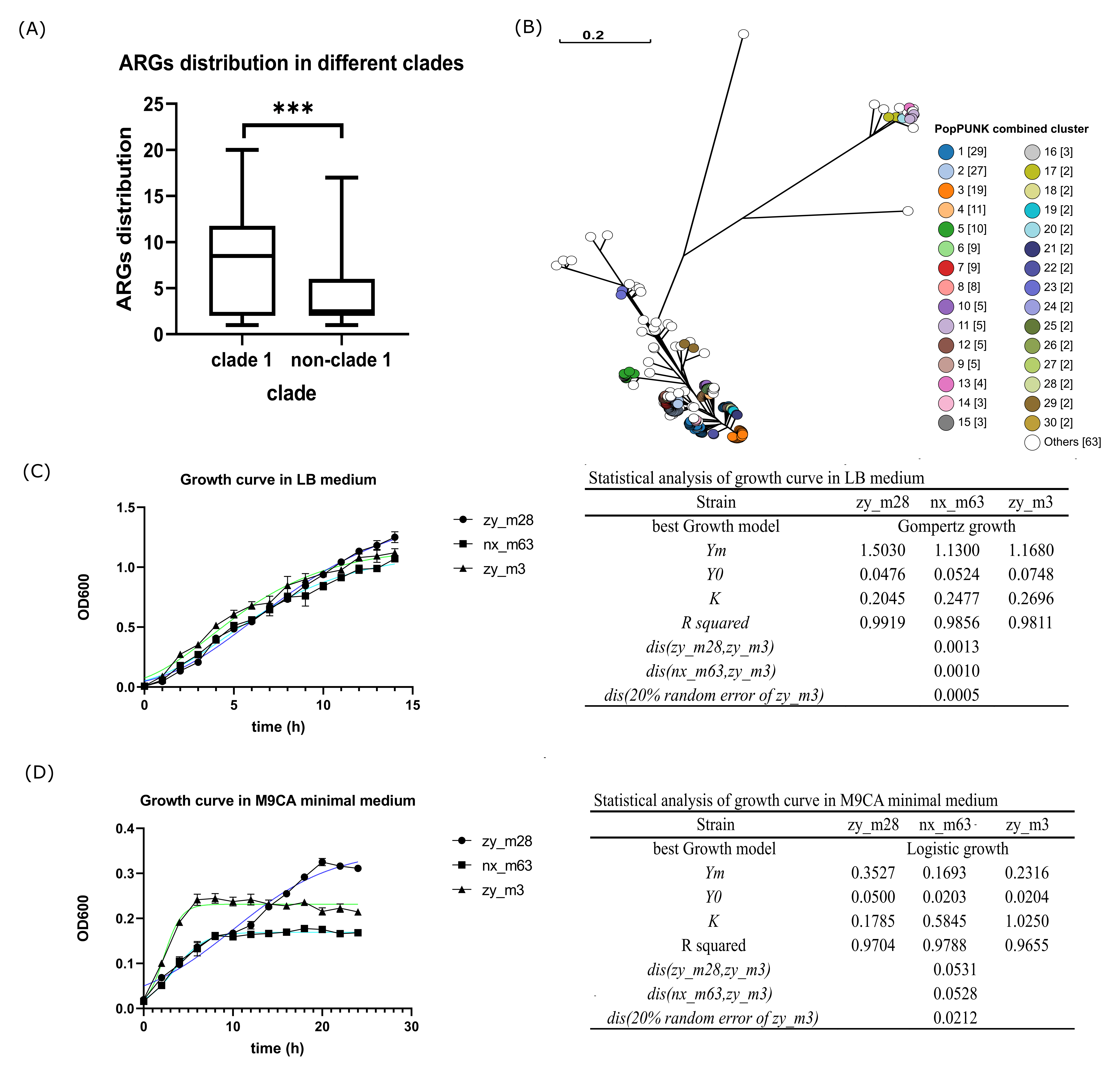
Figure S3** **(A)** Box plot of antimicrobial resistance genes distribution in different phylogenomic clades. Differences in characteristics between groups were analyzed using Mann-Whitney U rank sum test. **(B)** Population structure of *M. morganii* isolates by PopPUNK analysis. The picture is drawn by GrapeTree. Each circle represents an isolate and each line is a branch. Branch length varies on the distance between isolates. **(C)** Growth curve of *Morganella morganii* strains in LB medium. The colored smooth curve represents the fitted growth curve. **(D)** Growth curve of *Morganella morganii* strains in M9CA minimal medium. The colored smooth curve represents the fitted growth curve.


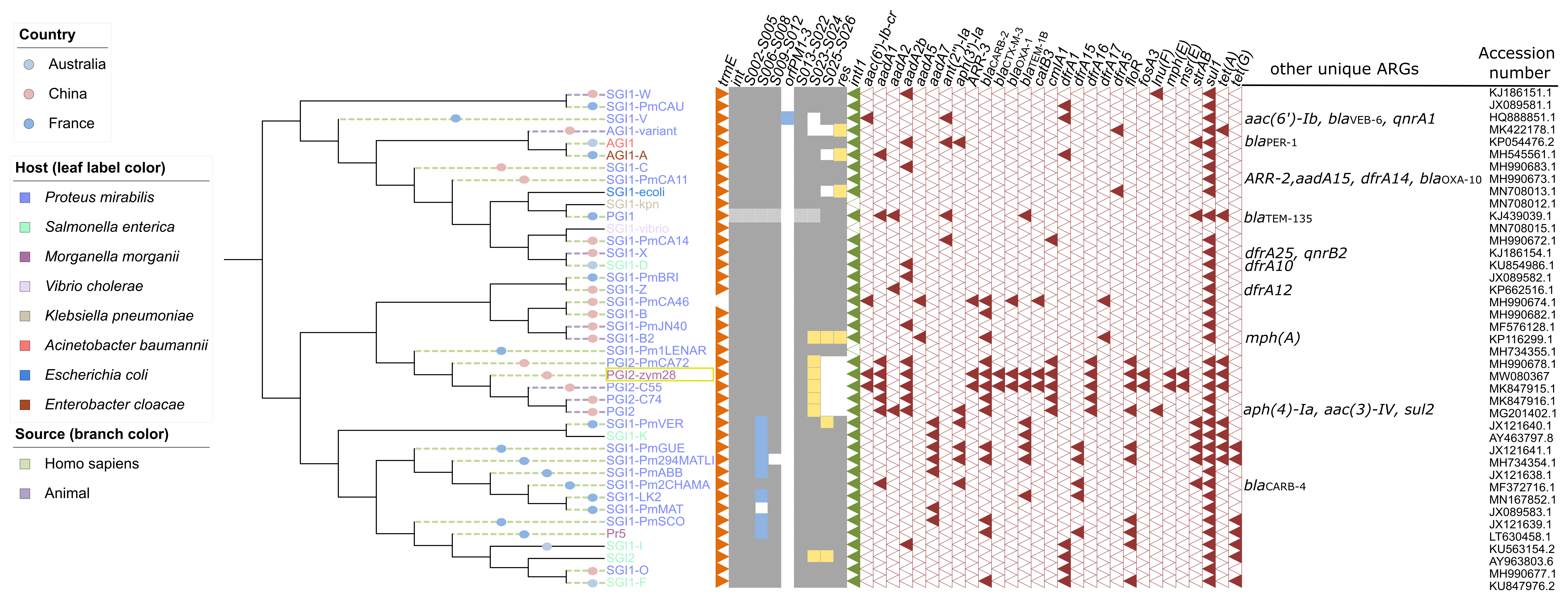


**Figure S4** Phylogenetic analysis of *Salmonella* related genomic islands combined with genetic context comparisons. Branch symbols represent the geographic origins. The color of dashed branches represents the isolation source. Bacterial hosts are indicated by the color of leaf labels. PGI2-zym28 in this study is noted with a yellow hollow rectangle. Filled dark gray rectangles represent genomic island backbones sharing more than 80% similarity to SGI1 backbone, while filled light gray rectangles represent genomic island backbones sharing less than 80% similarity to SGI1 backbone. Filled blue rectangles represent transposase insertion. Filled yellow rectangles represent inversion events. White rectangles represent deletions. Antimicrobial resistance genes are shown with filled red triangles.
